# Breaking Stereotypes: A Unique Presentation of New-Onset Multiple Sclerosis

**DOI:** 10.7759/cureus.47584

**Published:** 2023-10-24

**Authors:** Kyrillos Girgis, Jacob Brown, Kevin Lipat, Jose Bustillo

**Affiliations:** 1 Internal Medicine, Newark Beth Israel Medical Center, Newark, USA; 2 Internal Medicine and Pediatrics, Newark Beth Israel Medical Center, Newark, USA

**Keywords:** atypical presentation, focal neurological deficits, transient ischemic attack, acute stroke, multiple sclerosis

## Abstract

Multiple sclerosis (MS) is a chronic demyelinating disorder resulting in demyelination, neuroaxonal degeneration, and sclerosis. This often-debilitating disease affects young females mainly. Literature describing the pathology and phenotypic features is vast. Although there are extensive descriptions of new-onset MS presentations, few document the initial presentation as a transient ischemic attack or ischemic stroke. The case we present highlights the rarity of such presentation. In the literature, we found scarce reports about MS as presenting as a stroke mimicker with some studies quoting from 2.2% to 4.4% of the cases having MS. Our case serves as a reminder that MS can mimic acute ischemic strokes and the importance of maintaining MS apart of the differential in a young female with no significant history present with acute neurological deficits to reduce the complications of MS and the healthcare-associated costs.

## Introduction

Multiple sclerosis (MS) is a chronic demyelinating disorder in which lymphocytes attack myelin antigens, resulting in plaques of demyelination, neuroaxonal degeneration, and sclerosis. MS typically affects the white matter where myelin and oligodendrocytes are present [1]. Epidemiologically, the prevalence of MS is 30.1 per 100,000 persons [2]. MS commonly affects females more than males, specifically a 2-3:1 ratio with a mean onset of age 28-31 at onset [3-5]. MS can often be phenotypically variable, resulting in the presentation of atypical symptoms that mimic other disease processes making this diagnosis of MS challenging. The following case highlights the uncommon variability in disease presentation, specifically the rare presentation of new-onset MS mimicking an acute ischemic stroke [6,7].

## Case presentation

A 34-year-old female with no past medical history presented to our emergency department with acute onset of left upper and lower extremity weakness associated with numbness and tenderness of the left hand. The onset of symptoms occurred at 2 a.m., which she brushed off and went back to sleep. At approximately 7:30 a.m., she noticed that the weakness progressed to the point of falling. According to the family present during the incident, the patient had no slurred speech, facial drooping, or ataxic gait, but decided to bring her to our emergency department due to progressive muscle weakness. Before this episode, she described increasing depression over the last couple of months with no inciting factor.

**Figure 1:**
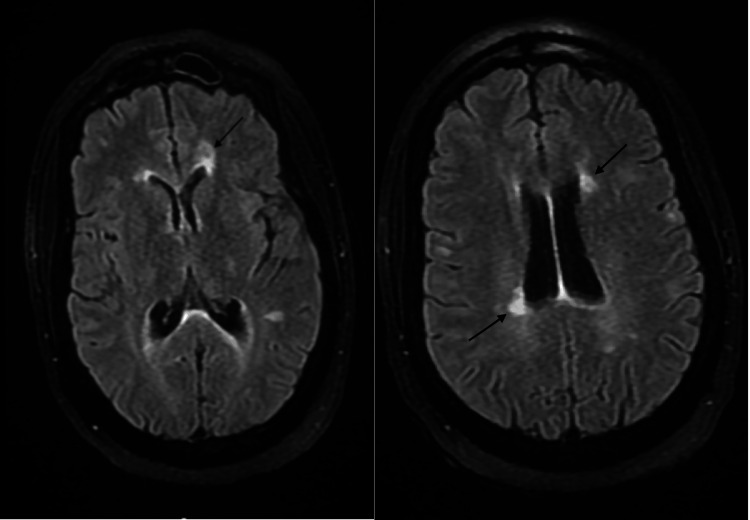
MRI brain T2 FLAIR showing multiple periventricular demyelinating lesions (black arrows) FLAIR: fluid-attenuated inversion recovery

**Figure 2:**
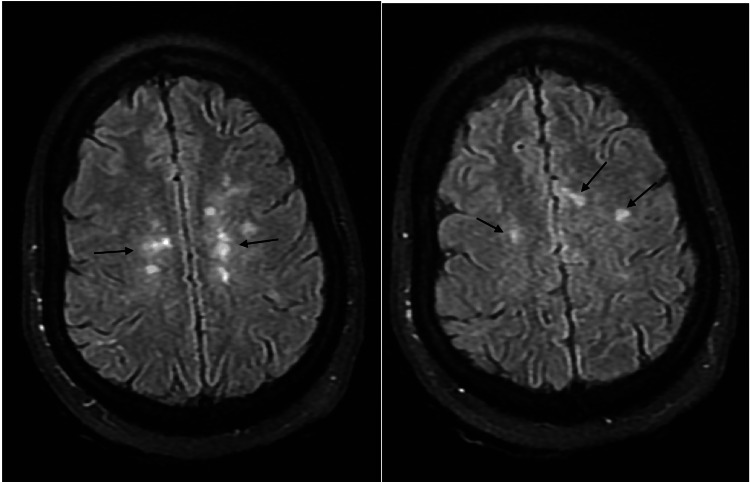
MRI brain T2 FLAIR showing multiple sub-cortical demyelinating lesions (black arrows) FLAIR: fluid-attenuated inversion recovery

**Figure 3:**
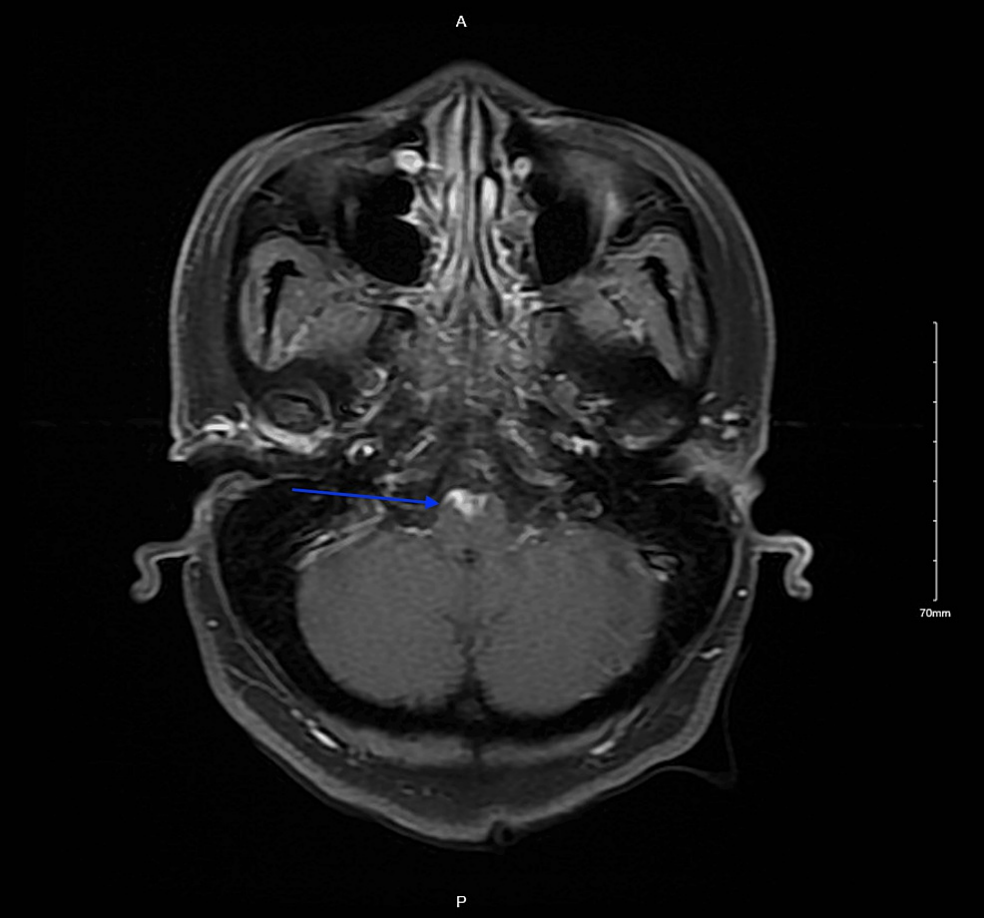
MRI brain with contrast showing anterior inferior pontine enhancement (blue arrow)

**Figure 4:**
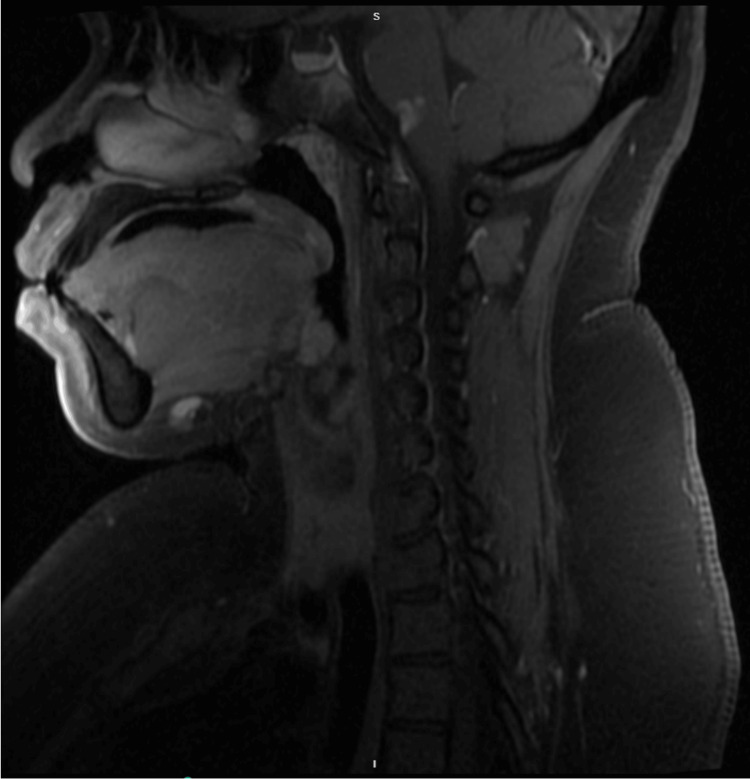
MRI of cervical spine showing no abnormal enhancement

Approximately 14 hours later, the patient presented to our emergency room. Vital signs in the emergency room were a temperature of 97.9°F, blood pressure of 136/99 mmHg, heart rate of 86 beats per minute, respiration rate of 18, and oxygen saturation of 99% on room air. The patient was alert, oriented, and in no acute distress on the physical exam. The initial neurological exam was negative for any signs of gross cranial nerve deficits, including facial droop or hemineglect. The sensation was intact throughout the upper and lower extremities. Muscle strength was 2/5 in the shoulder in all directions, elbow flexion and extension, wrist flexion, hip flexion and extension, knee flexion and extension, and dorsiflexion and extension. Right-sided strength was 5/5 in all planes. Neurological reflexes were within normal limits. The patient had abnormal dysdiadochokinesia and finger to nose on the left but normal on the right. Initial laboratory data showed a white blood cell count (WBC) of 6.9, hemoglobin (Hgb) of 12.8, and platelet count of 338. The comprehensive metabolic panel (CMP) was within normal limits with a thyroid stimulating hormone (TSH) of 3.060. The urine drug screen was negative. Given the patient's initial history and presentation, with increasing blood pressures in the 180s/100s, acute ischemic stroke or transient ischemic attack (TIA) was suspected, so a computed tomography (CT) head without contrast was completed and subsequently negative for both ischemic and hemorrhagic stroke. The patient was then subsequently admitted to the medicine service for further workup. While initially, differential diagnosis was comprehensive and included TIA vs. stroke, seizure, Todd's paralysis, complex migraine, and MS; MS became more likely when subsequent evaluations showed improvement in her neurological and strength examinations. During the hospital course, our patient underwent further evaluations with CT angiography of the head and neck, a 2D echocardiogram of the heart, an MRI brain, and an MRI spine. CT head and neck were negative for arterial stenosis, and the echocardiogram was negative for patent foramen ovale (PFO). MRI brain (Figure 1 and Figure 2) was done showing multiple fluid-attenuated inversion recovery (FLAIR) hyper-intense lesions in the subcortical white matter, periventricular, and medulla suggestive of areas of demyelination/MS versus less likely vasculitis. MRI brain with contrast (Figure 3) was then performed showing an area of parenchymal enhancement within the anterior inferior pons at the pontomedullary junction and along the medullary pyramidal tracts. MRI of the cervical spine (Figure 4) showed no abnormal signal or enhancement in the cervical spine.

Given the patient's neurological symptoms and multiple demyelinating lesions on the MRI brain with the presumed diagnosis of MS, a lumbar puncture was performed and CSF analysis was positive for more than five gamma restriction bands not found in serum. CSF also showed slight pleocytosis. The patient was then discontinued on aspirin and statin for stroke prophylaxis and started on pulse-dosed Solu-Medrol 250mg every six hours for 11 doses. Approximately six hours after initiating pulse steroid therapy and approximately 30 hours from symptom onset, her left-sided flaccid paralysis resolved, and she was appreciated to have 5/5 strength in the shoulder, elbow, wrist, knee, and ankle in all directions. Given the patient's rapid improvement of symptoms after initiating steroids and clearance by physical therapy, the patient was discharged home to follow up with neurology and her primary care physician.

## Discussion

Since 1996, there have been four established subtypes of MS based on the clinical findings, which included relapsing-remitting MS, primary progressive MS, secondary progressive MS, and progressing relapsing MS. In 2013, these four subtypes expanded to explore potential biological and imaging correlates. As a result, two new MS subtypes were incorporated: radiologically isolated syndrome and clinically isolated syndrome (CIS) [8].

CIS is a term used to describe an initial episode of neurologic symptoms that lasts for at least 24 hours due to inflammation or demyelination in the central nervous system (CNS). CIS is described as mono-focal (a single neurological deficit) or multifocal (more than one neurological deficit) [9]. CIS carries a risk of progression to MS, especially if brain lesions detected by MRI accompany the CIS. The difference between CIS and MS is that the latter fulfills the McDonald criteria of dissemination in time and space, while CIS is just a single episode of MS-like symptoms [10].

According to the 2017 McDonald criteria for diagnosing MS, dissemination in space means the presence of demyelinating lesions in at least two out of four regions of CNS, which include the spinal cord and three regions of the brain, which include the periventricular, the juxtacortical or cortical, and the infratentorial regions [10]. Dissemination in time means that neurological injury occurs at more than one point, which can be demonstrated by another episode of disease exacerbation or by MRI with gadolinium contrast which can differentiate between old and new lesions, as the new active lesions will be seen as gadolinium-enhancing lesions. In addition, the presence of oligoclonal bands in the CSF is evidence of CNS inflammation. Therefore, if they are present, the patient also meets McDonald criteria of dissemination in time [10].

Our patient's presentation can be classified as multifocal CIS and subsequently meets the criteria for MS. The dissemination in space was evidenced by multiple areas of demyelination in the brain and the spinal cord, as shown by the MRI. Moreover, the dissemination in time was illustrated by the MRI, which revealed active enhancing and old non-enhancing demyelinating lesions. Also, CSF oligoclonal bands on lumbar puncture fulfilled the dissemination in time criteria.

Most of the cases of MS present with acute optic neuritis described as loss of visual acuity, impairment of light perception, and color vision. Spasticity is also a common symptom due to the demyelination of the upper motor neurons. Other symptoms include fatigue, ataxia, and tremors [11]. Uthoff phenomenon is also common, a symptom exacerbation with exposure to high temperature [12]. On the opposite end of the spectrum, MS can rarely present as a mimicker of other diseases, such as acute ischemic strokes [6,7,13,14]. Risk factors associated with stroke mimickers are young age and women like the case presented above [11]. According to one study by Hosseininezhad M and Sohrabnejad R, who reviewed the chart of 1985 patients presenting to Poursina Hospital with an acute stroke, found that 295 (14.9%) patients had stroke-mimicking symptoms, and of those patients, only 4.4% had MS [12]. Another study conducted by Peking Union Medical College showed that out of 520 MS patients, only four cases, or 0.77%, presented with TIA or acute stroke-like symptoms [15]. These studies above show that MS mimicking acute stroke is a documented but rare phenomenon.

Therefore, this paper serves as a reminder that MS rarely presents as an acute weakness and should be considered in the differential diagnosis in a young female with no significant risk factors presenting with neurological deficits. Maintaining this slight suspicion can prevent patients from unnecessarily receiving systemic thrombolysis and the risk of significant bleeding.

## Conclusions

Since MS was discovered, there have been abundant studies describing initial presenting symptoms such as optic neuritis, spasticity, fatigue, ataxia, and even tremors, but little literature describing its presentations as acute ischemic stroke or TIA. This case serves as a reminder that young females with no significant medical history presenting with acute neurological deficits may be masking the underlying diagnosis of MS, leading to early treatment and acute outcomes.
